# Climate change impacts on small pelagic fish distribution in Northwest Africa: trends, shifts, and risk for food security

**DOI:** 10.1038/s41598-024-61734-8

**Published:** 2024-06-03

**Authors:** Abdoulaye Sarre, Hervé Demarcq, Noel Keenlyside, Jens-Otto Krakstad, Salaheddine El Ayoubi, Ahmed Mohamed Jeyid, Saliou Faye, Adama Mbaye, Momodou Sidibeh, Patrice Brehmer

**Affiliations:** 1 ISRA, Centre de Recherches Océanographiques de Dakar-Thiaroye, CRODT, BP 2241, Dakar, Sénégal; 2 MARBEC, IRD, Ifremer, CNRS, University Montpellier, Sète, CS 30171, Avenue Jean Monnet, 34203 Sète cedex, France; 3 Geophysical Institute, University of Bergen and Bjerknes Centre for Climate Research, Bergen, Norway; 4 Institute of Marine Research, P.O. Box 1870, 5817 Nordnes, Bergen, Norway; 5 INRH, Institut National de Recherche Halieutique, Casablanca, Maroc; 6 Institut Mauritanien de Recherche Océanographique et des Pêches, IMROP, BP 22, Nouadhibou, Mauritanie; 7 Fisheries Department, FD, Marina Parade, Banjul, The Gambia; 8 IRD, University Brest, CNRS, Ifremer, LEMAR, SRFC, CSRP, Dakar, Senegal

**Keywords:** Small pelagic fish, Climate change, Spatial shifts, Sea surface temperature, Upwelling intensity, Fisheries, Food security, Northwest Africa, Ecology, Biooceanography, Climate-change ecology, Ecosystem ecology, Tropical ecology, Marine biology, Physical oceanography

## Abstract

Climate change is recognised to lead to spatial shifts in the distribution of small pelagic fish, likely by altering their environmental optima. Fish supply along the Northwest African coast is significant at both socio-economic and cultural levels. Evaluating the impacts of climatic change on small pelagic fish is a challenge and of serious concern in the context of shared stock management. Evaluating the impact of climate change on the distribution of small pelagic fish, a trend analysis was conducted using data from 2363 trawl samplings and 170,000 km of acoustics sea surveys. Strong warming is reported across the Southern Canary Current Large Marine Ecosystem (CCLME), extending from Morocco to Senegal. Over 34 years, several trends emerged, with the southern CCLME experiencing increases in both wind speed and upwelling intensity, particularly where the coastal upwelling was already the strongest. Despite upwelling-induced cooling mechanisms, sea surface temperature (SST) increased in most areas, indicating the complex interplay of climatic-related stressors in shaping the marine ecosystem. Concomitant northward shifts in the distribution of small pelagic species were attributed to long-term warming trends in SST and a decrease in marine productivity in the south. The abundance of *Sardinella aurita*, the most abundant species along the coast, has increased in the subtropics and fallen in the intertropical region. Spatial shifts in biomass were observed for other exploited small pelagic species, similar to those recorded for surface isotherms. An intensification in upwelling intensity within the northern and central regions of the system is documented without a change in marine primary productivity. In contrast, upwelling intensity is stable in the southern region, while there is a decline in primary productivity. These environmental differences affected several small pelagic species across national boundaries. This adds a new threat to these recently overexploited fish stocks, making sustainable management more difficult. Such changes must motivate common regional policy considerations for food security and sovereignty in all West African countries sharing the same stocks.

## Introduction

In this study, our primary focus is on sardinella (*Sardinella aurita* and *Sardinella maderensis*) because of their paramount importance for food security^1–3^. These small pelagic fish have long been a vital resource for citizens of the northwest African region and a crucial part of fisher livelihoods^4^. However, the situation has become increasingly challenging over the past two decades^3,5^. Fishers report a concerning northward and offshore shift in the distribution of sardinella. Concurrently, there has been a decline in the abundance of sardinella and the impoverishment of local fishing communities^6^.

This decline in sardinella is linked to the intensification of fishing efforts during our study period from 1995 to 2015^3,7^. In contrast, in some other regions, fishing pressure and forage fish biomass are less related^8^. In some cases, the long-term fluctuations in small pelagic fish stocks have instead been attributed to variability in recruitment success, primarily influenced by oceanographic conditions during the juvenile stage^9–11^.

The consequences of climate change across different domains present challenges in anticipating and integrating them into policy and management planning^12–14^. There are uncertainties surrounding future emission scenarios, as well as climate change and its complex impacts. These uncertainties are acute in marine ecosystems and fisheries, especially in the highly productive Eastern Boundary Upwelling Systems^15–17^. Dealing with these uncertainties is particularly challenging for developing countries, where access to data is limited.

The CCLME benefits from seasonal upwelling, which contributes significantly to the recruitment and abundance of small pelagic fish^11,18^. However, only between 21 and 26° N is upwelling quasi-permanent. *Sardinella aurita*'s annual migration in northwest Africa closely aligns with the seasonal dynamics of the coastal upwelling in the sub-region^19–21^. Until the 1970s, the northern limit of *S. aurita*'s migration was considered to be around 24° N^22^. In Senegal, young *S. maderensis* individuals tend to congregate between Gambia and Guinea Bissau during January–February, with a high concentration of young spawners observed in this area through March and April^23^. In July, they migrate towards Dakar and further northwards, reaching their northernmost position around November before starting their southward migration in December-January. Regarding the migration of adults *S. maderensis*, it is understood that they leave the Senegalese coast and can be found along the coast of Mauritania from February to September and near Cape Blanc from October to January.

A thorough understanding of the critical elements that impact the productivity of pelagic habitats for major commercial fish species, as well as the typical oceanographic patterns within the CCLME, has been gradually emerging^15,24,25^. However, our understanding of their spatial and temporal variations remains limited^20,26,27^. The shifts in the large-scale distribution of small pelagic resources were probably affected by factors beyond just changes in where fishing activity occurred. An alternative explanation of these distributional changes is that the environmental changes have exceeded the pelagic ecosystem's homeostatic capacity, leading to long overdue changes^39^ in the structure of its populations. Indeed, the capacity of an ecosystem to support the population dynamics of a given species heavily relies on its physical and biological conditions as key determining factors.

The goal of this paper is to investigate and assess the impact of climate trends on the spatial distribution of small pelagic fish, particularly focusing on sardinella along the coast of Northwest Africa.

## Materials and methods

### Study area

**Figure 1:**
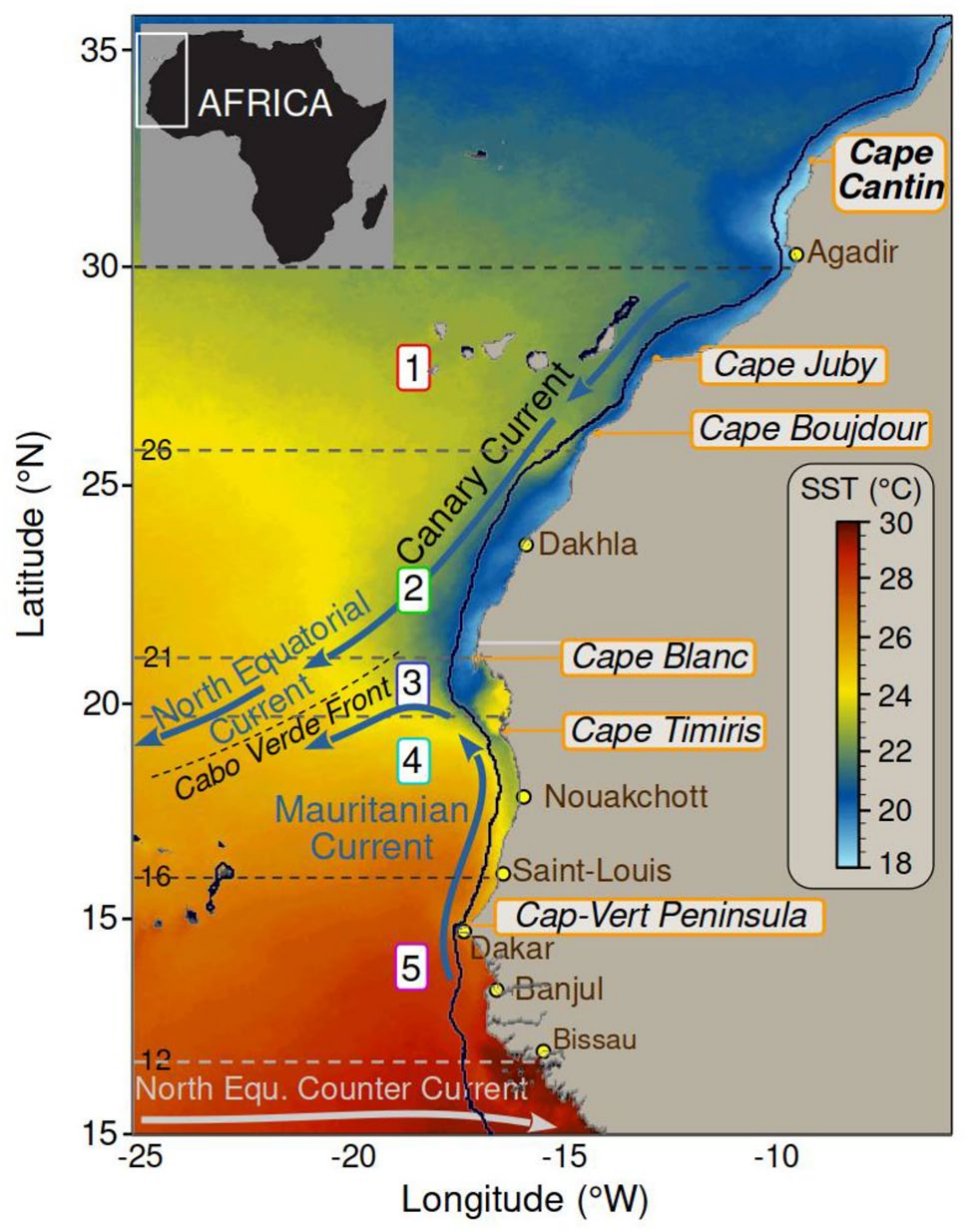
Map of the southern Canary Current Large Marine Ecosystem in North West Africa showing the Sea Surface Temperature (SST) for November 1995–2015. Main currents are superimposed: the Canary Current, the North Equatorial Current, the Mauritanian Current and the Cabo Verde Frontal Zone. The region was divided into five areas (dotted lines): (1) North of Cape Boujdour [30–26° N], (2) between Cape Blanc and Cape Boujdour [26–21° N], (3) around Cape Blanc [20–21° N], and (4) the Mauritanian [21–16° N] and (5) Senegalese [16–12° N] areas. Software IDL 7.1.

The study area is the south part of CCLME, characterised by high biological productivity^15,28^. Our focus is directed from South Morocco (26° N) to Guinea (10° N). Several North West African countries share the same small pelagic fish stocks within this East Border Upwelling system where no concerted regional management occurs^29^. These stocks play a key role in the national economy of several states and are of primary importance for food security. In this study, we divide the region into five upwelling areas: North of Cape Boujdour, between Cape Blanc and Cape Boujdour, around Cape Blanc between 20 and 21° N, and the Mauritanian and Senegalese areas^28,30^ (Fig. 1).

### Ecological environment of small pelagics in Northwest Africa

In the Eastern Central Atlantic, there are three distinct regions where *S. aurita* is especially abundant^31^, all characterised by coastal upwelling. In this region, *S. aurita* inhabits the continental shelf in non-turbid and salty waters, with temperatures typically below 24 °C^32,33^. Other studies mention the coast of Morocco as the northward limit of distribution of *S. aurita*^34,35^. Recently, a genetic study showed a significant differentiation detected between the northernmost specimens (Morocco to Guinea) and the southernmost ones (Liberia to Angola)^36^. *Sardinella aurita* may withdraw below the thermocline in the warm season and be found at depths of 200 m at night^37^, whereas schools occupy surface waters during the day^38^. *Sardinella maderensis*, in contrast, is euryhaline and lives in the coastal zone, often associated with estuaries and water temperatures above 24 °C^39^. This species is considered less sensitive to environmental changes^40^.

*Sardinella aurita* along the Northwest Atlantic coast migrates seasonally between upwelling convergence zones^21,41^. The first adults pass the peninsula of Cap-Vert (Senegal) in January and concentrate between Dakar and Guinea-Bissau, where they spawn and stay until April before starting their northward migration as the surface waters warm up. After spawning, the juveniles leave the nursery area in southern Senegal and join the adults' seasonal migrations. They reach their northernmost distribution, north of Cape Blanc (24°–25° N), in November to mid-December. The adults scatter from October to December off Mauritania and soon migrate southward from January to February.

*Sardinella maderensis* has a less pronounced migratory behaviour than *S. aurita*^42^. It is reported to be relatively sedentary^43^, with movements in Senegal and Mauritania confined to the nursery areas. The Senegalese-Mauritanian stock of *S. maderensis* was distributed along the coast approximately from 26° N to 10° N.

Sardinella occurs with several other exploited small pelagic species of less socio-economic interest at the time of our study. Although our primary focus rests on the two sardinellas (see paragraph 2.1), our examination also encompasses *Trachurus trecae* (Cunene horse mackerel), *Caranx rhonchus* (False scad), *Selene dorsalis* (African moonfish), *Chloroscombrus chrysurus* (Atlantic bumper), *Brachydeuterus auritus* (Bigeye grunt) and *Sphyraena guachancho* (Guachanche barracuda)*.* These species are commonly found on the shelf (< 100 m depth) in the tropical CCLME region^26,44^ and are often caught together with the sardinellas. Adult *T. trecae* and *C. rhonchus* leave these shoals and are commonly found in deeper, colder oceanic water masses^16,45^.

### The West African hydroacoustics surveys

Small pelagic fish species were monitored through annual hydroacoustic surveys performed by the R/V Dr Fridtjof Nansen (Norway), referred to hereafter as DFN. These surveys were carried out annually from November to December 1995 to 2006 and then in 2011 and 2015 (Table S1). The surveys covered the region from Casamance (12°16' N) in Southern Senegal to Cape Cantin (32°30' N) in Morocco (Fig. 1).

The DFN database was evaluated, and the extensive sampling spanning 170,000 km and 2263 fishing operations were analysed to determine potential shifts in the latitudinal distribution of small pelagic fish and their relationships to changing environmental conditions. The northern latitudinal extreme locations where the species were reported were observed from trawl catches, while their spatial distributions were determined from acoustics data^46^.

The DFN surveys always went well beyond the northernmost distribution of both sardinella stocks, except for the 2005 survey, which stopped in Morocco at 26°18' N due to technical problems. Thus, for the 2005 survey, *S. aurita* and *T. trecae,* distributed north to 26°18' N, were not computed. All the surveys were specifically designed to assess the small pelagic fish stocks, except for the 2011 survey.

In 2011, DFN survey spacing between transects was 20 nm; consequently, there is a larger uncertainty around the distribution than during the previous acoustic biomass surveys. Thus, it was decided not to compute the biomass indexes, as their accuracy is less than during ordinary surveys. The sampling protocol was otherwise consistent throughout the time series, with high sampling intensity (a total of 2263 trawls performed from 1995 to 2015; Table 1) and using the same fishing gears, i.e., two "Akrahamn" pelagic trawls^47^ and one "Gisund super bottom trawl"^48,49^. The trawl doors used for all trawling operations were Thyborøn type 7 "combi doors" (weight 1 720 kg and surface area of 7.4 m^2^). Distributional maps of pelagic fish and the acoustic biomass were based on data recorded by a Simrad 38-kHz transducer connected to a scientific echosounder (Simrad EK 500 and from 2007 EK60;^49^). The echosounder was calibrated using standard techniques^50^, constant echosounder parameters, e.g., 20 log R time varied gain function (R is the range, in m) and 1 ms pulse length. The data were post-processed using the Bergen Echo Integrator^51^ and the LSSS software (2011 and 2015 surveys)^52^.

**Table 1:** Number of trawls and occurrences (%) of the eight species considered in the study from all trawl sampling stations (n = 2 263) from 1995 to 2015.

Year	Nb of Trawls (2263)	Occurrence of species in trawls (%)							
		S_aur	S_mad	C_chr	D_rho	B_aur	S_dor	T_tre	S_gua
1995	158	43	36	19	32	28	16	27	20
1996	129	35	26	16	29	20	12	26	9
1997	115	47	41	22	23	20	13	26	10
1998	155	37	30	17	28	17	14	32	8
1999	164	38	34	20	23	21	23	38	7
2000	133	28	31	26	35	26	27	38	20
2001	115	41	45	26	29	26	20	24	18
2002	185	30	28	16	22	15	15	44	10
2003	175	30	30	17	22	19	14	38	10
2004	158	34	36	25	31	22	18	32	8
2005	131	39	34	30	25	28	23	43	24
2006	155	35	37	20	21	22	18	41	14
2011	281	14	7	8	19	17	12	19	9
2015	209	17	18	11	23	22	12	22	10
Mean	162	33	31	19	26	22	17	32	13

Species abbreviations: S_aur = *Sardinella aurita*; S_mad = *Sardinella maderensis*; C_chr = *Chloroscombrus chrysurus*; D_rho = *Caranx rhonchus*; B_aur = *Brachydeuterus auritus*; S_dor = *Selene dorsalis*; T_tre = *Trachurus trecae*; S_gua = *Sphyraena guachancho*. All surveys were performed during November (Table S1 for the detailed date). The surveys always went well beyond the northern distributional limit of the sardinella, apart from the 2005 survey, which stopped in Morocco at 26°18' N.

### Methodological approach

#### Biological data processing

The small pelagic species are selected due to their commercial importance, their sub-tropical or tropical affinity, and their frequent occurrence in the scientific trawl catch samples between 1995 and 2015 (Table 1) and in the DFN surveys (Fig. 1). Eight pelagic species followed these criteria: both sardinella species (*S. aurita* and *S. maderensis)* and six others: *T. trecae*, *C. rhonchus*, *S. dorsalis*, *C. chrysurus*, *B. auritus,* and *S. guachancho.*

To detect spatial changes in pelagic fish distributions, the time series of scientific trawls are analysed to extract the annual northern limit (maximal North latitude), i.e., where these species are caught in the DFN trawls database. This "northern limit" is defined as the northernmost location where each species is present in the DFN trawl catches. To estimate how far north each species has moved, we compare the yearly changes in latitude using data averaged from equalised surveys conducted during two time periods: 1995–2001 (7 surveys) and 2002–2015 (7 surveys). The Northward shift is computed by determining the difference between the northern limits of each species for the two periods, expressed in km per decade (Table S2). To assess the significance of these differences, we use a bootstrap procedure involving 10,000 random re-samplings (Boot package, R version 4.2.2). Additionally, linear trends for each species' northern limit are analysed over the entire time frame from 1995 to 2015. The significance of these trends is evaluated using the Pearson correlation coefficient. This approach allowed us to study the Northward distribution shift for each species.

All the DFN surveys used in this study were carried out during November–December, with a high and consistent sampling intensity (Table 1). A bootstrap procedure is later used to test the temporal change of *S. aurita* proportion of acoustic biomass north and south of Cape Blanc between 1995–2001 and 2002–2015.

#### Environmental data and analysis

Historical remote sensing data sets were compiled and analysed to assess sub-regional changes in key environmental variables over the 21-year biological dataset. Additionally, these datasets enabled monitoring of significant environmental shifts since the initial records in 1982, thereby updating previous studies^28,53^ and facilitating a detailed understanding of environmental dynamics and providing valuable insights into long-term trends, enhancing our ability to monitor and manage environmental changes.

While short-term environmental fluctuations may lead to rapid distributional responses^54^, long-term changes can create cumulative effects that alter the broader habitat characteristics, impacting small pelagic fish distribution patterns on longer time scales. This broader perspective allows us to develop a more comprehensive assessment of the factors shaping the habitats of these ecologically important species.

Fish habitats are defined by a set of biophysical parameters such as sea temperature and primary production, all structured by the intensity of the coastal upwelling system. To identify potential drivers for changes in the distribution of the small pelagic fishes, consideration is given to alterations in (1) sea surface wind speed (SWS), (2) the wind-based Ekman upwelling index, (3) sea surface chlorophyll-*a* concentration (SSC), and (4) sea surface temperature (SST). All environmental variables were extracted for the five areas defined above (Fig. 1).

Two distinct periods were considered for the analysis of environmental times series SST, SSC, SWS, and upwelling index. The first is tied to the timeframe of the fish sampling, encompassing the annual acoustic sea surveys conducted by DFN from 1995 to 2015, i.e., the biological sampling survey period, referred hereafter to BSS. The second period spans a more extended duration and is designed to examine long-term environmental changes, referred to hereafter LEM. This analysis encompasses data from as early as 1982 for SST, 1998 SSC, and 1988 for SWS and the upwelling index, extending up to 2021.

The SSTs were extracted from the AVHRR pathfinder V5.3 dataset (1982–2021) (adapted from^55^ at a spatial resolution of 1/24° of latitude and longitude and a daily resolution [NOAA National Centers for Environmental Information. Dataset^56^]. The SWS data were extracted from the daily CCMP (Cross-Calibrated Multi-Platform available at www.remss.com) wind product V2.0 at the 0.25-degree spatial resolution from 1988 through 2021. The Ekman upwelling index is computed as in^57^ from the SWS dataset, except that the coastline angle, used as a guideline for the computation of the wind stress, is replaced by the 200 m isobath angle. The SSC, a proxy of the primary productivity^58^, was collected from both the SeaWiFS (1998–2010) and MODIS sensors (2011–2021) at the exact resolution as the SST data set (1/24° and daily), both available from https://oceancolor.gsfc.nasa.gov/cgi/browse.pl. All data were corrected and cross-calibrated during the common period 2003–2010, as described in^59^, aiming to mitigate observed biases between both sensors. All these data (SWS, SST, and SSC) were compiled by month and year depending on their further use, respectively, for display purposes and the computations of trends, for which yearly averages are entirely adequate.

All satellite data were calculated for the five coastal areas defined (Fig. 1) at a standardised distance of 100 km from the coast. This spatial selection aimed to enhance the precision of environmental assessments on coastal upwelling, focusing on capturing and quantifying the impacts on sea surface temperature (SST) trends. Temporal trends in the time series were computed using the least square linear adjustment method, applied to both environmental and biological datasets. The primary emphasis of this paper is on trends that implicitly consider shift effects, exemplified by the shift in SST observations in 1994–95 and for both the SWS and the upwelling index in 1998, notably North of Cape Blanc (areas 1–3). For visual representation through maps, the time series were segmented into distinct periods, i.e., the LEM period and the BSS period. The respective averages for each period were then calculated to facilitate a comprehensive understanding of the trends. This method demonstrates robustness and typically yields results like those obtained through linear trends. The similarity is often assessed by minimising root mean square differences or, in some instances, absolute differences (called robust fitting). Further discussions on this approach can be found in^53^. Last, linear trends were performed for each environmental variable describing the hydro-climatic conditions for both BSS and LEM periods and tested using the Pearson correlation coefficient.

## Results

### Changes in the distribution of sardinella

**Figure 2:**
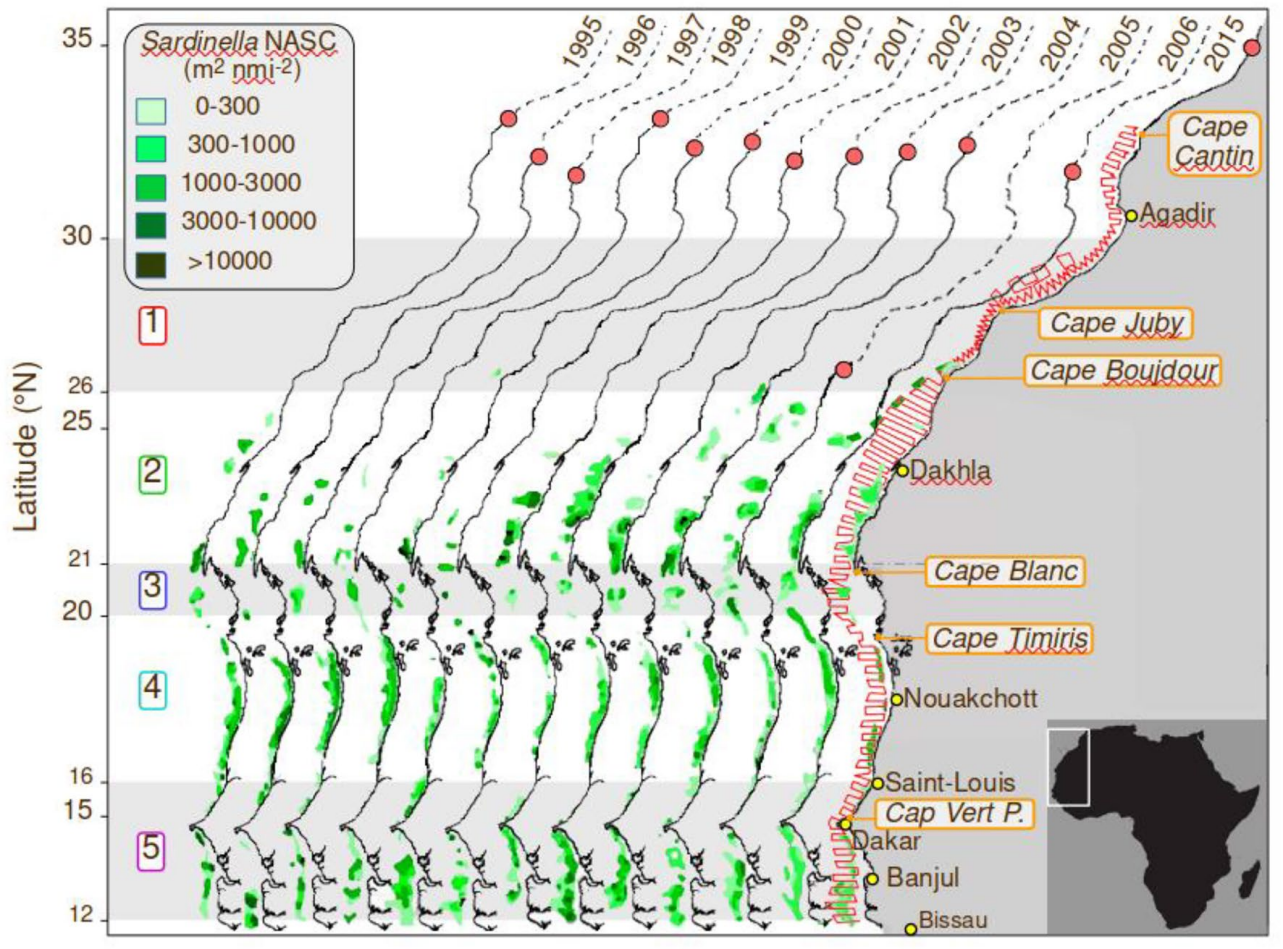
Distribution of both sardinellas (*Sardinella aurita* and *S. maderensis*), extending from Cape Roxo (Senegal) to Cape Boujdour (Morocco), from 1995 to 2015, based on annual acoustics surveys with DFN. Fish density (nautical area backscattering coefficient 'NASC' expressed in m^2^ nmi^−2^) is indicated by different intensities of green. The northern limit of detection, proven by fishing operations, is shown as a dark green line. The northernmost latitude covered by the surveys is presented by a red circle each year. A red line presents the typical design of the acoustic surveys. This distribution includes both sardinella species, with *S. aurita* being the main contributor north of 20° N. The numbering on the left, in colour, represents the five areas used for hydroclimatic partitioning (see Figs. 1 and 4). Sotfware IDL 7.1 and LibreOffice 24.2.

**Figure 3:**
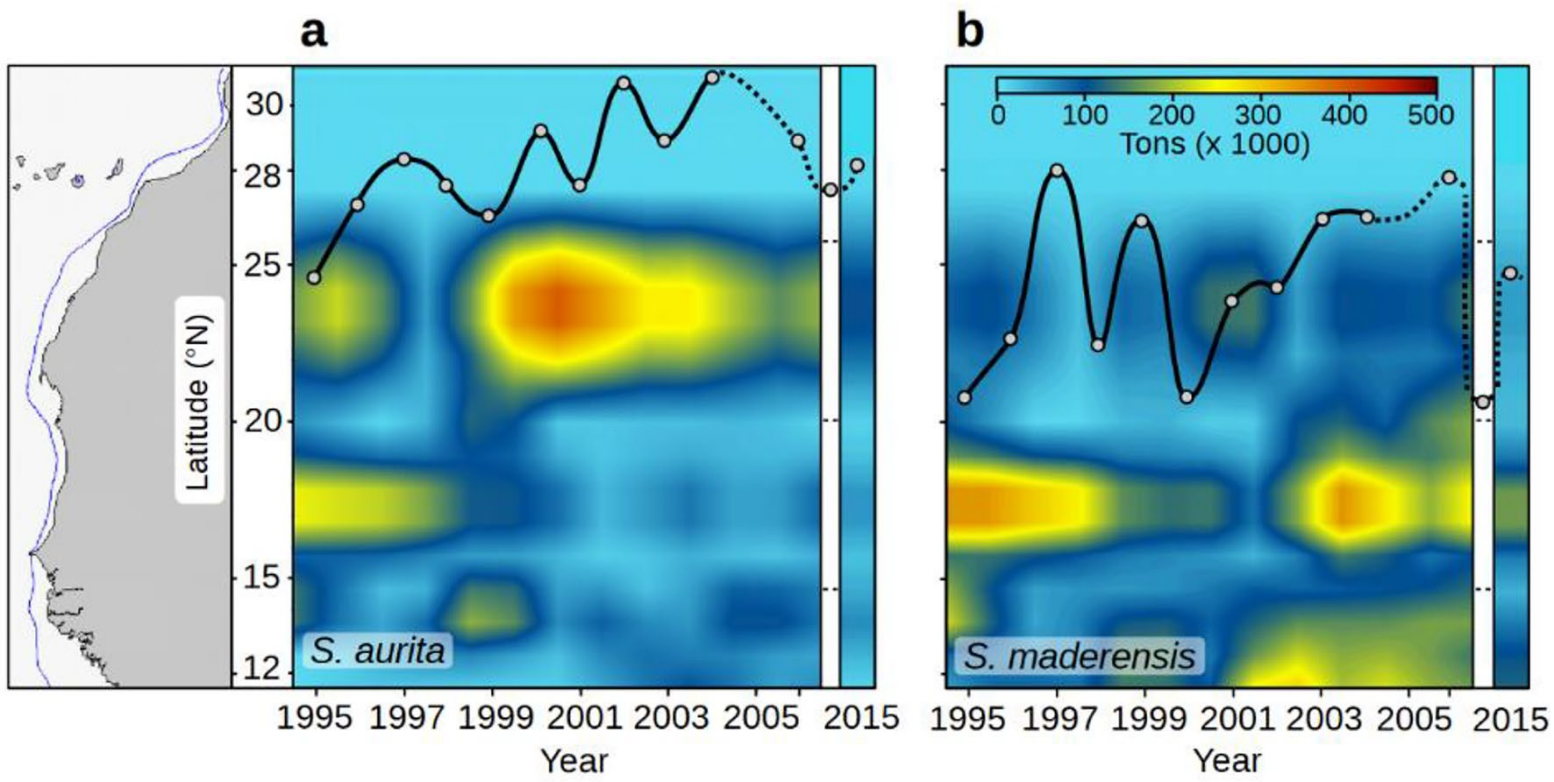
Hovmoller representation of the spatio-temporal changes in the spatial distribution of *Sardinella aurita* (**a**) and *S. maderensis* (**b**) during annual acoustics surveys in their distribution area (map on the left; Fig. 2). The northernmost detection of both species is plotted as a black line as detected by the 2 363 scientific trawl operations. The dotted sections of the lines represent periods without year-to-year surveys. Annual biomass is expressed in thousands of tons. The white area represents the 2011 survey without an acoustic biomass estimate. The blue line on the map shows the 200 m isobaths. Sotfware IDL 7.1 and LibreOffice 24.2.

From the acoustic-based biomasses of both sardinella, only *S. aurita* displayed a northward shift during the BSS period (Fig. 2), while *S. maderensis* displayed a quasi-stable distribution over the same 20-year period (Fig. 3).

Moreover, a similar significant (*p* < 0.01) displacement of *S. aurita* northern limit computed from trawl catches is reported. This northern limit has moved northward at a significant rate of 181 km per decade since 1995 (Table S2).

### Changes in the distribution of other species

**Figure 4:**
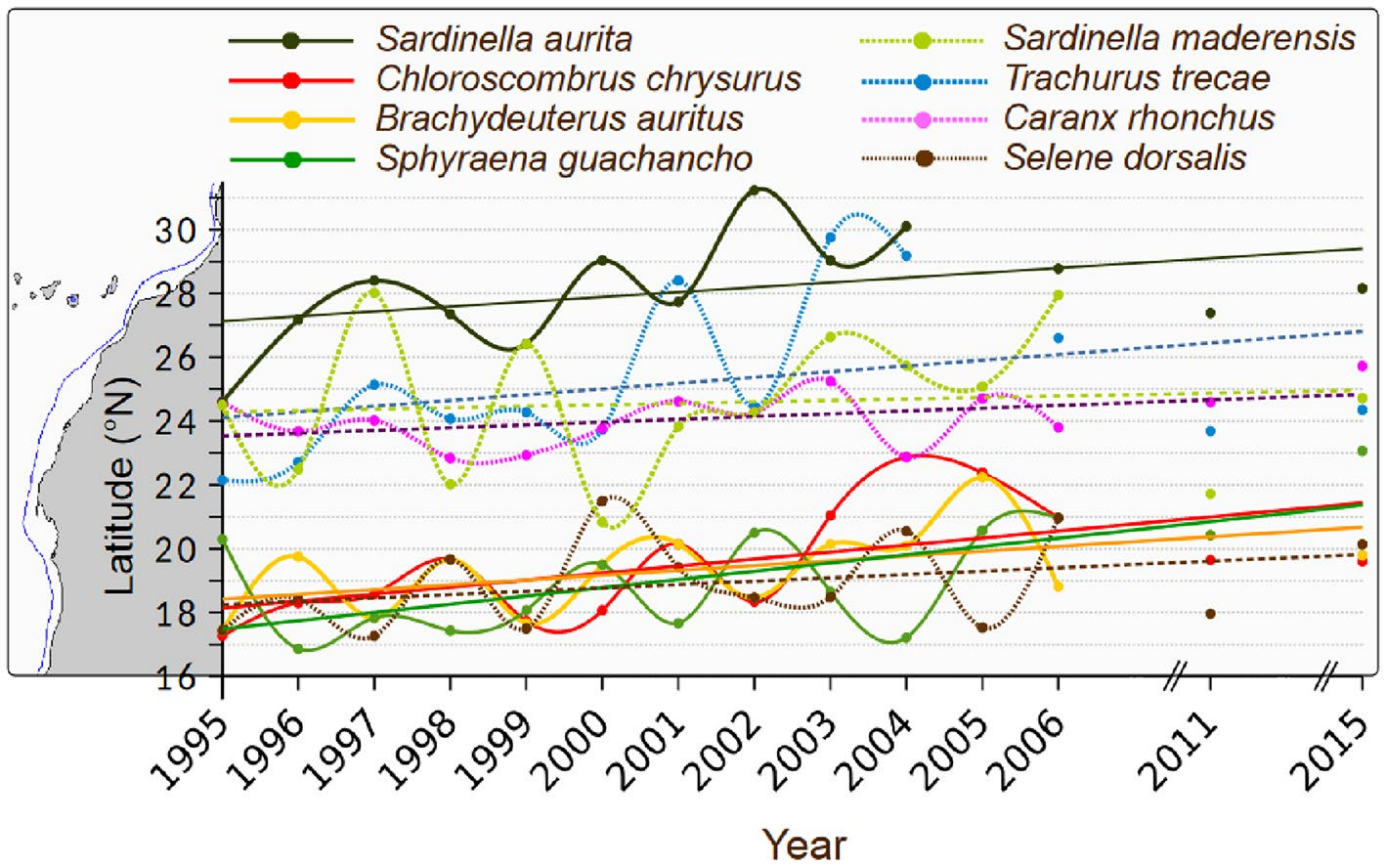
Changes in the distribution limits of the pelagic stocks of eight species sampled during the acoustic surveys from 1995 to 2015. Their northernmost limits were determined based on scientific trawl sampling data (n = 2 263). Solid lines represent significant trends (*p* < 0.05), while dotted lines indicate non-significant trends (see Table S2 for values). Sotfware IDL 7.1 and LibreOffice 24.2.

Other species than *S. aurita* also showed significant northward distribution shifts (Fig. 4 and Table S2): *Trachurus trecae* moved regularly towards the north from 1995 to 2004 and then stopped its extension so that by the end of the observation period, its limit was found around 26° N. The northern limit of *C. chrysurus* was around 18.5° N in 1995 and progressively moved northward to 20.4° N in the most recent years; this represents a shift in the distribution of 195 km per decade (Table S2). No acoustic estimate was available for these species.

The distribution shift of *B. auritus* showed a slower but regular and, therefore, very significant trend of 118 km per decade. *Brachydeuterus auritus* was not found north of 19° N before 2000 in the surveys but was reported five times after 2000. Similarly, the distribution area of *S. guachancho* gradually extended northward after 1995 (Fig. 4), when its northern limit was south of 18° N, and then expanded northwards to 21° N in 2005–2006, a considerable shift of about 200 km per decade. The false scad (*C. rhonchus*) and *B. auritus* showed the slowest significant northward shift of the species considered in this study, with a speed of 70 km per decade. *Sardinella maderensis,* a tropical species, did not display any significant (*p* > 0.05) distributional shift (Fig. 4), and *S. dorsalis* displayed a reasonably stable distribution area (Table S2). A shift distance close to 200 km per decade was observed for three species (*T. trecae*, *S. guachancho,* and *C. chrysurus*) showing similar northward extensions of their ranges of two hundred km per decade, a value precisely of the same order that of *S. aurita*.

### Changes in hydro-climatic conditions

The southern part of the CCLME exhibits the greatest surface warming ever recorded from observations among all tropical Oceans (Fig. S1), and the trend, computed for the 1982–2021 period, reaches 0.3 to 0.5 °C per decade.

#### Environmental trends over the biological sampling survey period

**Figure 5:**
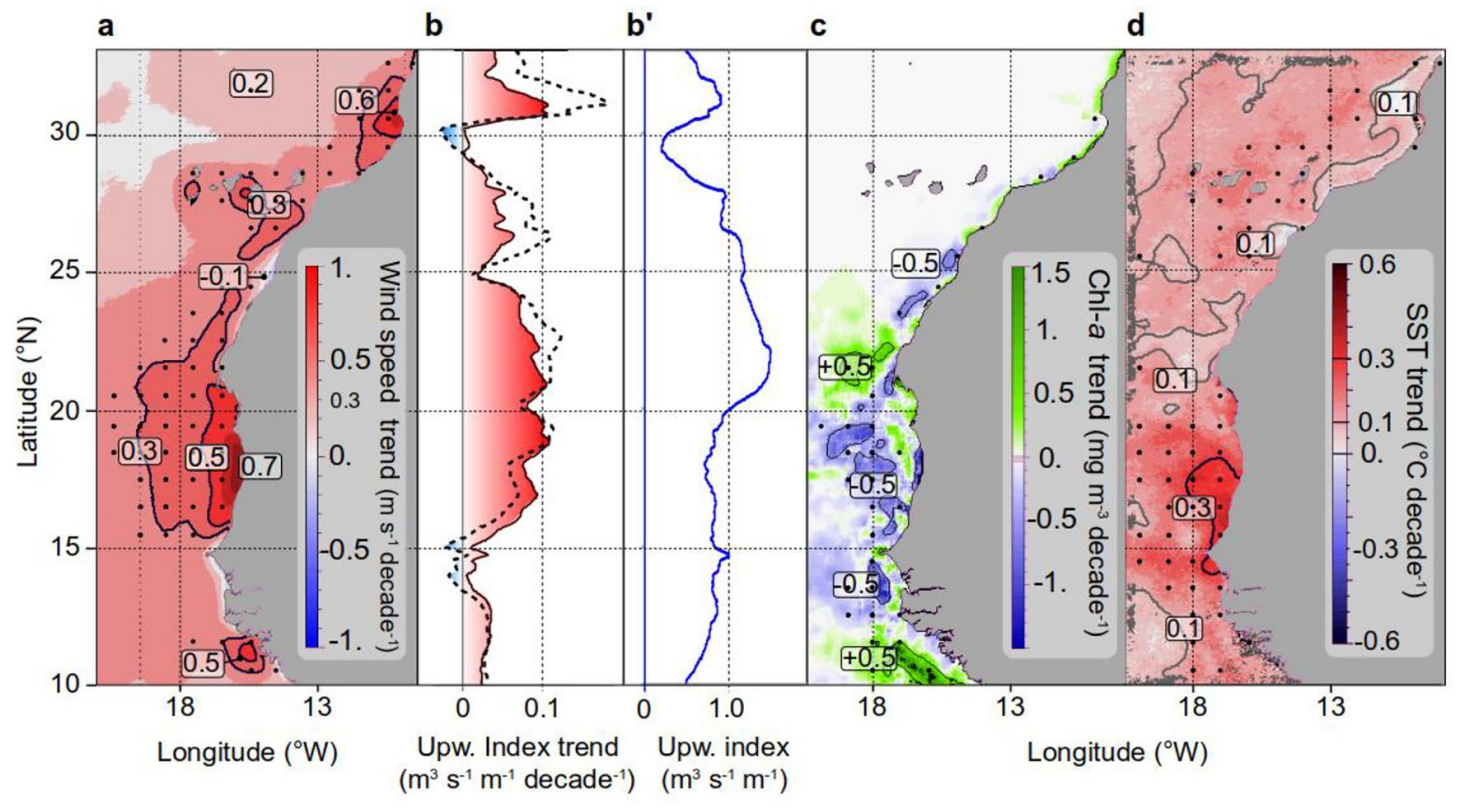
Spatial linear trends in hydro-climatic conditions in North West African coastal waters from 1995 to 2015 of (**a**) the wind speed; (**b**) the upwelling index (see “Method” section); b') the latitudinal profile of the upwelling index as a reference; (**c**) The chlorophyll-*a* concentration from SeaWiFS and MODIS-corrected data (computed from 1998; and **d**) the sea surface temperature (SST) from AVHRR. Black dots show significant trends (*p*-value < 0.05). The trend of the upwelling index for the entire 1988–2021 period is illustrated with a dotted line in panel b, superimposed to demonstrate temporal homogeneity in the upwelling increase compared to the 1995–2015 period. Sotfware IDL 7.1 and LibreOffice 24.2.

During the 20-year sea survey period, both trends in SWS and upwelling index were still highly significant (Table S4, right column), particularly in the coastal domain in Mauritania (area 2) (Fig. 5a,b). The upwelling index showed the same positive significant trend from North to South during the sea survey period (Fig. 5b, solid line) as reported during the most extended time series (Fig. 5b, dotted line).

**Figure 6:**
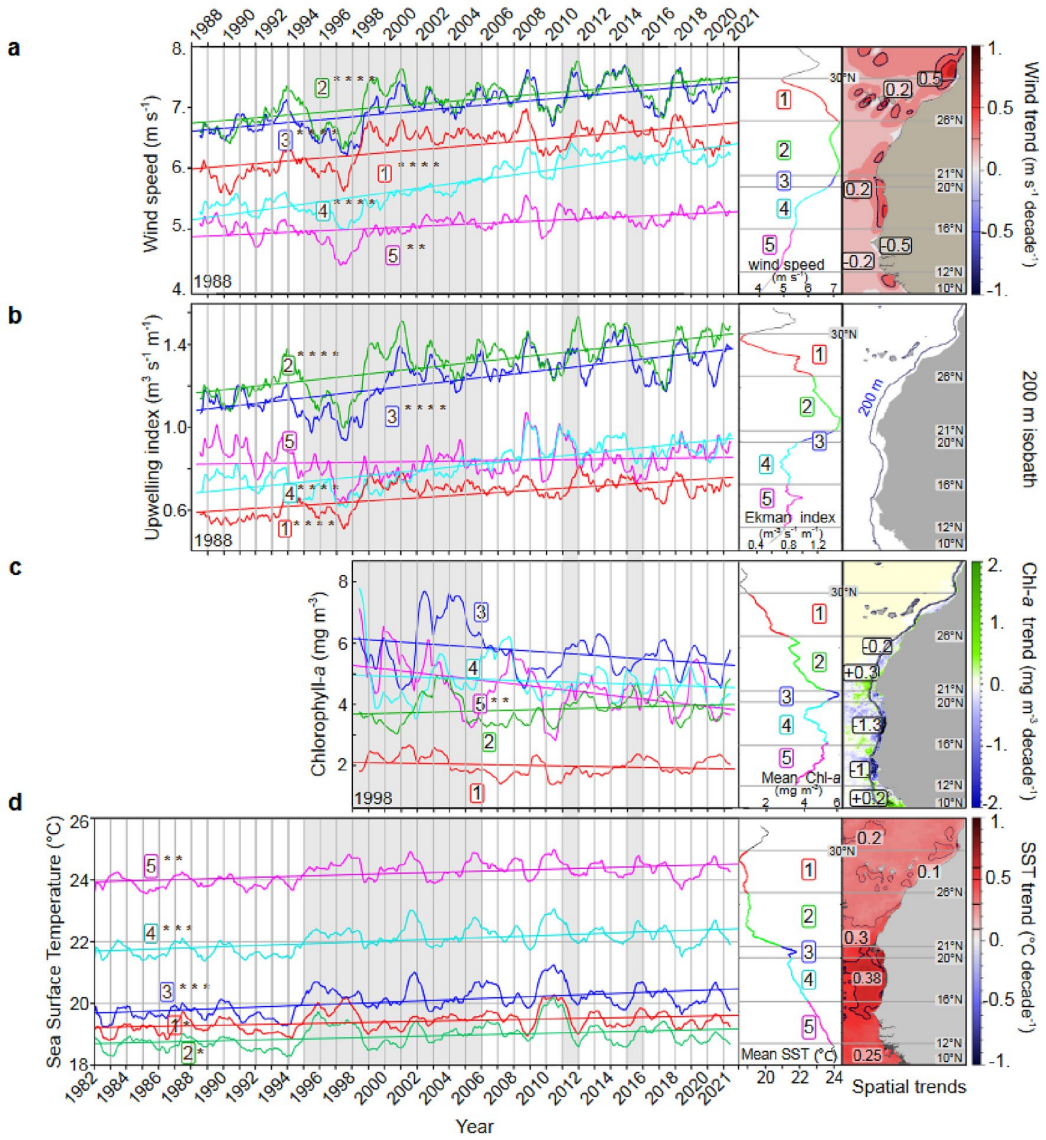
Changes in hydro-climatic conditions along the Northwest coast of Africa for five latitudinal areas (numbered 1 to 5 from North to South, see Fig. 1). Monthly averages (12-term moving averages) of (**a**) the wind speed and (**b**) upwelling index since 1988 (from CCMP satellite composite data); (**c**) the chlorophyll-*a* concentration (SeaWiFS and MODIS-corrected data since 1998); and (**d**) sea surface temperature since 1982 (AVHRR data) from Morocco (area 1) to Southern Senegal (area 5) until 2021 (four decades). Least square linear adjustments of trends are indicated by area and significance level (*p*-value: * > 0.05, ** > 0.01, *** > 0.001, and **** > 0.0001). All data were spatially averaged from the coast to 100 km offshore. The middle plots represent the average latitudinal profile of the time series. Maps (right panels) show the main values of the spatial trends per decade. The periods covered by the biological sampling survey (1995 to 2006, 2011, and 2015) are shaded in grey. Yearly aggregated data are provided as the supplementary Table S5. Sotfware IDL 7.1 and LibreOffice 24.2.

The temperature data analysis reveals a lack of consistent and intense warming trends. A notable shift in SST occurred in 1994–95 (Fig. 6d), with no discernible trends observed before or after this period across all regions. Although some localised spatial trends were significant (Fig. 5d), they failed to demonstrate consistency on broader scales. Moreover, the warming pattern does not exhibit uniformity or progression over time, making linear trends unsuitable for capturing abrupt changes accurately.

#### Spatial variability on hydro-climatic conditions over the long environmental monitoring period

Since 1988, highly significant intensification of the upwelling intensity was registered (Figs. 6b and 5b-b'; Table S4) north of Cape Blanc (20° N) (areas 1 to 3; Fig. 6) as well in Mauritania (area 4). On the contrary, the Senegalese area showed a strong drop up to 1999, followed by a second phase of moderate but regular increase. Accordingly, the SWS increase was highly significant in all areas from 1988 to 2021 (34 years; Fig. 6a).

The SSC showed no significant changes in any area (Fig. 6c and Table S4 and S5) due to their high and contrasting variability on shorter temporal scales except in Senegal (area 5), where a very significant negative trend was observed. Areas 3 and 4 displayed remarkably high and variable average levels of SSC between 4 and 8 mg m-3, the highest in the CCLME (Fig. 6c), while the very coastal northern Morocco (area 1) was remarkably stable.

SST displayed a regular and homogeneous intense warming trend throughout the three southern areas (Fig. 6d and Table S4), while moderate but significant warming was observed North of Cape Blanc (areas 1 to 2). The increase was maximum between the peninsula of Cap-Vert and Cape Blanc with a local pattern of ~ 0.4 °C decade^−1^ during the past 40 years (Fig. 6d, right panel). The SST increase was weaker in the northern part (0.1 to 0.3 °C decade^−1^).

**Figure 7:**
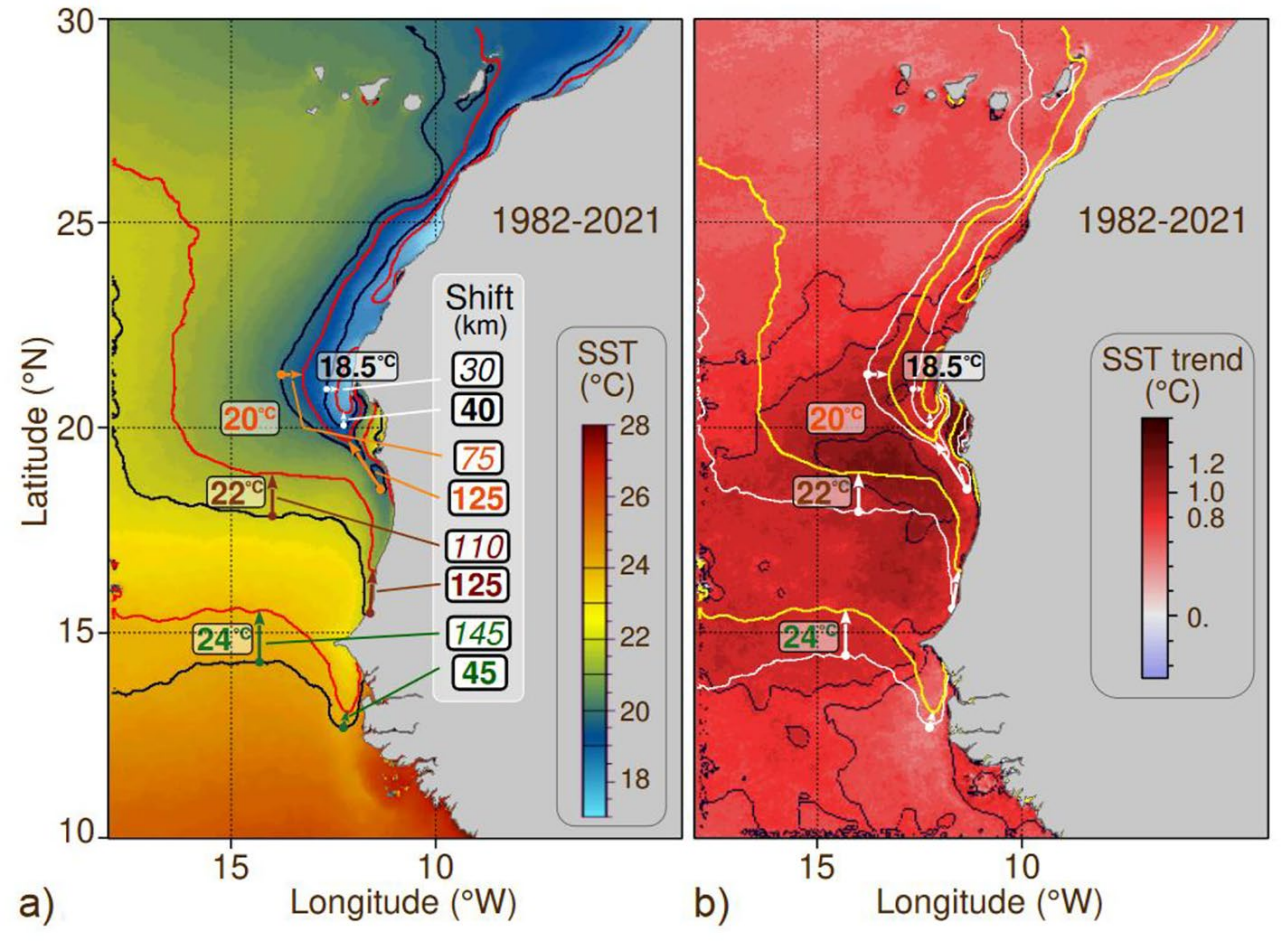
Spatio-temporal shifts of sea surface isotherms in the southern Canary Current Upwelling System (1982–2021). (**a**) The black and red lines represent the average positions of isotherms 18.5, 20.0, 22.0, and 24.0 °C during the 1982–2001 (20 years) and 2002–2021 (20 years) periods, respectively. The background colours show the average sea surface temperature (SST) from 2002 to 2021. Coloured arrows illustrate the displacements of isotherms between both periods, distinguishing between coastal and offshore domains. The central "shift" frame provides the displacement distances in kilometres. (**b**) The corresponding SST anomaly (in °C) between both 20-year periods. Contoured black lines at 0.8, 1.0, and 1.2 °C represent SST anomaly values and offer insight into temperature variations' magnitude and spatial distribution over the analysed time frame. These data are reported in Table S5. Sotfware IDL 7.1 and LibreOffice 24.2.

In the southern coastal CCLME, from 1982 to 2021, the impact of global warming was illustrated differently by the northward displacement of isotherms (Fig. 7; Table S3), reported as the average move in 20 years. The 24 °C isotherm moved northwards off Senegal at a rate between 45 and 145 km per decade, according to the distance to the coast. The 22 °C isotherm moves faster, especially close to the coast, at a rate of 125 km in 20 years with similar offshore displacements. Closer to the permanent upwelling region, the 20° isotherm moves at a similar speed at the coast, while its offshore zonal displacement was less important (75 km). This tendency continues for the 18.5 °C isotherm, close to the upwelling centres of the permanent upwelling region, i.e., areas 3 and 4, with displacements of 40 and 30 km, respectively.

All trends were tested for each of the four yearly time series of environmental variables, and each latitudinal area (Fig. 6). Most of them were highly significant, as detailed hereafter. The SST trends recorded during the BSS period did not show significant warming when aggregated by area. Nonetheless, high values of 0.3 °C decade^−1^ were locally recorded off central southern Mauritania and Senegal (Fig. 5d) despite stronger SWS in Mauritania.

SWS and upwelling index displayed a considerable increase in the whole region from 1988 to 2021 (see Fig. 4 for plots and maps and Table S4 for a statistical test of trends), except for the upwelling index in Senegal (area 5), where overall stability was observed during this period.

Over the longest time series, SSC trends along the sea survey period showed no significant trend, except in Senegal (area 5), where a significant decrease (Fig. 6c; Table S5) was observed. This decrease was associated with the stability of the upwelling intensity (Fig. 6b) and persistent sea warming (Fig. 6d).

## Discussions

### Northern spatial shift of small pelagic

Whatever the period considered, the Cape Blanc region (Area 3) appears to be a transitional area regarding SST (Fig. 5d), separating a southern part (Areas 4 and 5) characterised by a regular increase in SST and lower and stable upwelling intensity, and a northern part experiencing a continuous increase of the upwelling intensity. Over the LEM period, a significant SST anomaly (0.1 to 0.3 °C decade^−1^) was observed all year round, north and south of Cape Blanc (Fig. S1). During the BSS period (1995–2015), these anomalies were similarly weak from April to August and again in November, higher in September and October before dropping to negative values from December to March. Such changes showed that despite some interdecadal variability and a likely change in seasonality, the average warming was stable during the BSS period.

The northward shift in *S. aurita* distribution observed over the two decades of sea surveys was concomitant with the important catch increase recorded in Morocco at the end of the studied period in 2015^44^. Many marine species worldwide experienced a poleward shift in their population due to a shift in their thermal habitat^13,60^. We hypothesise that the exceptional SST warming observed from Mauritania to Senegal, the strongest ever recorded in the tropics during the LEM period (Fig. S1), induced similar displacements for several species. These facts could account for the exceptional presence of *S. aurita* up to Casablanca during the Moroccan-led small pelagic fish assessment in 2015. The DFN scientific catch data indicate a northward shift of 180 km for *S. aurita* along the studied period. The synergistic effects of climate variability and change on species fitness^61^ mediate small pelagic habitat suitability. It was well-established that short-lived species, like small pelagic fish, are highly sensitive to short-term environmental variations, and these fluctuations play a crucial role in shaping their distribution in the immediate term^62^. However, the significance of considering the impact of long-term environmental changes on small pelagic fish habitats must be recognised. Over time, gradual environmental modifications can influence these species' overall habitat suitability combined or not to the effect of overfishing.

Like *S. aurita*, five additional small pelagic fish species displayed significant northward trends in their distribution, while two others, including *S. maderensis,* exhibited no significant trend. As deduced from its acoustic-based biomass, *S. maderensis* displayed no significant shift in its northern limit and overall distribution. Given that all surveys were consistently conducted during the fourth quarter of the year, it is unlikely that the observed shifts are attributable to irregular survey timing or a regional increase in stock biomass^63^ (Fig. S2).

The assessment of latitudinal shifts in the fishery has previously employed calculating a monthly catch centre of gravity^64^. However, it is important to note that the use of catch rates per vessel as an index of fish abundance could be disputed. This is particularly true in Mauritania waters, where the fishery is primarily industrial. The catch rates depend on fish abundance and are influenced by catchability^65^ and socio-economic factors^6^. Some stochasticity must be expected in the observations from the sea surveys; this means that the species' northern limit was not necessarily observed. The records from DFN sea surveys were based on trawls carried out to identify all pelagic fish schools. The watch officer started scientific fishing operations only when significant acoustic densities of pelagic fish were observed. Consequently, low densities or fish presence inshore in the survey area have probably been overlooked^66,67^.

Any shift in the distribution of sardinella and other small pelagic stocks could lead to significant economic and social instabilities. In Mauritania and southern Morocco, fisheries are responsible for a significant part of the gross domestic product, particularly as major investments have been made in establishing fishmeal factories^68,69^ to process these species. Recently, the pressure on these stocks has increased because of the emergence of fish meal factories and the arrival of semi-industrial vessels, i.e., purse seiners and trawlers^5^. *S. aurita* is now especially overexploited^63^. In Senegal during the BSS period, no specific change in the focus on targeted species has been observed. Landings of sardinella remain the most important in terms of biomass, even though local authorities have supported a pronounced alternative policy in favour of fish farming over the past years. At the sub-regional level, amidst the diverse threats facing fish resources, a mounting apprehension within all national fisheries centres revolves around establishing a unified monitoring system for transboundary exploited fish, pre-empting the onset of significant fisheries conflicts^70^.

### Spatial considerations

The Cape Blanc boundary at around 21° N roughly corresponds to the position of the Cape Verde Frontal Zone^71^ that occurs in November off Mauritania and separates the North and South Atlantic Central Waters in the eastern North Atlantic Subtropical Gyre^72^. Until recently, this location has been regarded as a “faunistic limit” for many benthic^73^, planktonic^74^, and ichthyoplanktonic^75^ populations. It was also considered the northernmost distributional area for several small pelagic fishes in northwest Africa^39^. DFN surveys indicate that *C. chrysurus, B. auritus,* and *S. guachancho* were moving beyond this boundary in the second part of the LEM period.

The observed warming patterns appear to significantly influence the spatial restructuring of small pelagic fish populations, including *S. aurita*. Notably, the northern distances observed in the spatial shift of the pelagic fish (between 40 and ~ 200 km) align closely with the magnitude of the isotherm’s displacements (40–145 km) since 1995 (Fig. 6; Table S3). SST is likely a primary driver behind the movements of *S. aurita,* as suggested by previous studies^76–78^. It is important to note that the observed changes are not solely attributed to SST; various physical and ecological factors, including regularly increasing winds and decreased primary productivity south of Cape Blanc, contribute to this transformative shift.

**Figure 8:**
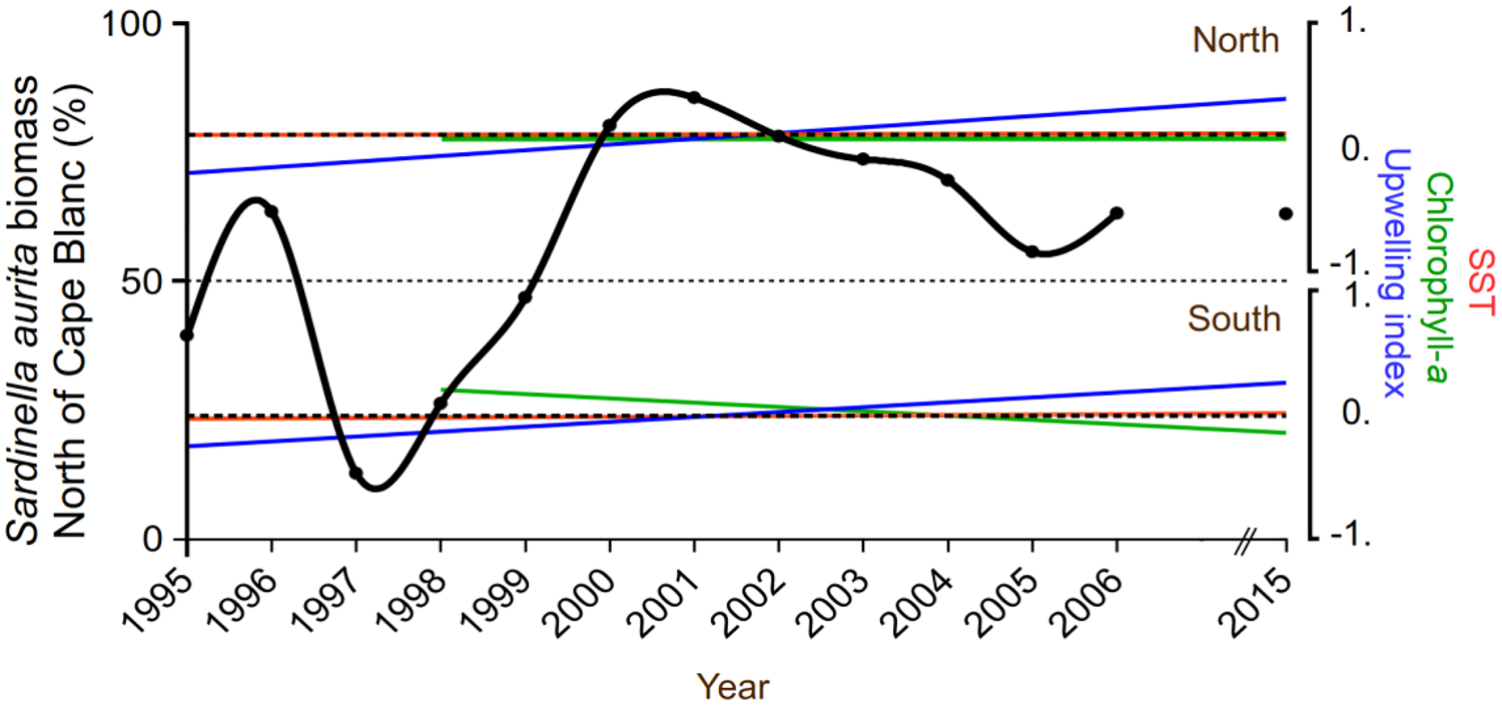
Relative variation of *Sardinella aurita* biomass and the main hydro-climatic conditions from 1995 to 2015 North and South of Cape Blanc—a pivotal zone separating the region of permanent upwelling from the subtropical region of seasonal upwelling. The percentage of *S. aurita* biomass north of Cape Blanc (black solid line) has increased in the recent part of the time series. The linear trends of the coastal upwelling index (blue lines), chlorophyll-*a* (green lines), and sea surface temperature (SST, red lines) exhibit an overall increase in upwelling conditions in both sectors. Additionally, a noticeable decrease of chlorophyll-*a* was observed south of Cape Blanc, while no significant SST trends were identified (see Table S5). Sotfware IDL 7.1 and LibreOffice 24.2.

The trend in *S. aurita* biomass showed that the portion of the stock north of Cape Blanc rose from 38 to 65% from 1995 to 2015 (Fig. 8), with a significant difference (*p* < 0.001) between the first and the last part of the time series. Notably, the northern part (Fig. 5b, area 1), characterised by a strong summer coastal upwelling^79^, was the region with one of the greatest increases in SWS intensity across the entire Canary Current ecosystem over the last 34 years, including the BSS period. To examine the hypothesis of a northern population displacement (Fig. 3a, Fig. 8) triggered by strong and/or rapid environmental changes, particularly the quick rise in SST observed in 1994–95 and the intensified upwelling in 1998 (Fig. 5b and 5d), we employed two distinct bootstrap procedures. Across all analyses, we consistently observed significant to highly significant changes in SWS and upwelling index in all areas. In contrast, no change was recorded for SCC except in area Senegal (area 5), and SST exhibited significant change only in the LEM period and not in the BSS one (Table S4).

While the entire CCLME was notably impacted by long-term warming (Fig. S1), the heightened upwelling in the northern part of the region (North of Cape Blanc) mitigates coastal heating and maintains productivity. Consequently, this area offers more favourable habitat conditions and a relatively stable thermal environment for small pelagics. In stark contrast, the southern part of the system (area 5, Senegal) experiences a modest increase in SST coupled with a decline in phytoplanktonic biomass (Fig. 5). This observation aligns with the findings of^80^ over a shorter period (2002–2016). It implies that this region is likely becoming a less productive habitat for some species.

The acoustic biomasses of *S. aurita* and *S. maderensis* during the BSS period (Fig. S3) showed that *S. aurita* biomass was decreasing, with the lowest value observed for the last year (2015). This decline has continued in recent years^63^. During these 21 years, the average SWS north of Cape Blanc was higher than 7 m s^−1^, well over the optimum environmental window of 5 m s^−1^ defined for the recruitment success of small pelagic fishes^11,81^. Therefore, environmental conditions are likely not favourable for all species according to their reproductive success^3^.

### Ecological responses

The absence of a significant shift in the distribution of *S. maderensis* might be explained by their high physiological adaptability to environmental disturbances^40^ and the absence of rivers or estuaries in the West Sahara (areas 2 and 3) providing a natural habitat for its life cycle. In contrast, *S. aurita,* while opportunistically taking advantage of primary enrichment^11,82^, is more sensitive to warming, making it less adaptable to strong and continuous changes in ocean temperature as reported south of Cape Blanc. This difference in habitat shift can be explained by the environmental sensitivity of *S. aurita,* which exhibited less phenotypic plasticity^83^ than *S. maderensis*^40^ and, therefore, more prone to spatial adaptation.

*Brachydeuterus auritus*, *C. chrysurus, S. dorsalis,* and *S. guachancho* are known to be present in brackish waters during their juvenile stage^84^. For this reason, their northern border of distribution remains mainly south of the Cape Verde Frontal Zone and notably further south than those of the other species considered in this study (Fig. 3) that thrive in the richest part of the coastal upwelling, beyond the transition zone between southern and northern CCLME. In particular, *S. dorsalis* did not move significantly. This species is considered semi-demersal, or "the least pelagic" among the eight selected in this study, with a diet based on zoobenthos as nekton^85^.

The spatiotemporal shift in low thermal tolerance species, such as *S. aurita*, off Mauritania appears primarily controlled by thermal constraints rather than water-enrichment gradients^1^. Alternatively, the shift in these species might be related to the expansion and raising of the oxygen minimum zone^86,87^, occurring on the shelf to the south of the system. Such changes in water temperature and dissolved oxygen levels might influence trophic relationships, leading to major changes in the composition of planktonic prey species. *Sardinella aurita* is an opportunist feeder^32^, primarily feeding on phytoplankton (mainly diatoms) in offshore waters^88^, but also copepods, which are the two most abundant phytoplankton and zooplankton groups in the study area. Any alteration in the plankton community can disturb the whole food web^89^, influencing the small pelagic fish community, their abundance, and distribution^90,91^. However, the observed shift in *S. aurita* beyond 26° N was not related to the primary production level, as there was no significant increase in SSC to the north of Cape Blanc (Table S4). The complex interplay between trends in SWS and planktonic productivity (Fig. 6b,c) underscores indirect biogeochemical processes and variations in planktonic compositions that likely vary seasonally, consistent with Bakun's theory of summer warming^92^. Identifying the determinism of the phenological shift is challenging, as SST increase can modulate population characteristics, predator–prey interactions, intra- and inter-specific interactions, and induce potential physiological changes in marine organisms^93^.

Like *S. aurita*, five other commercial species showed significant northward trends in their distribution, both in the DFN data and in the Moroccan acoustic surveys, which detected exceptionally high densities of small pelagic fish off Casablanca (33.5° N) in 2015^94^. Among them, *C. chrysurus*, *B. auritus,* and *S. guachancho* have been regularly observed north of 20° N since 2003. Spatial shifts in species assemblage within West Africa are likely to impact ecosystem composition, productivity, fishing endeavours, and national economies. With additional warming, it was hypothesised that species currently constrained by their limited northward distribution might expand their range further northward due to a scarcity of estuarine habitats. Consequently, an abrupt northward displacement in the distribution of both *S. maderensis* and *S. dorsalis* could be anticipated, along with the possibility of a stock collapse, given their reliance on brackish water during various life cycle stages. Based on recent observations (post-2016), there is a southward shift in sardine populations, with significant catches reported in the area south of Cape Timiris. While our study focused on the northward displacement of certain species due to warming, the observed southward shift in sardines suggests that other factors beyond temperature may be driving changes in distribution patterns. Possible explanations for this phenomenon could include shifts in ocean currents, changes in prey availability, or alterations in eggs or ichthyoplankton survival. Therefore, the complex interplay of various ecological factors must be considered when predicting future distribution shifts. Additionally, the observed southward movement of sardines highlights the need for ongoing monitoring and adaptive management strategies to address the dynamic nature of species responses to environmental change.

Finally, for the whole sub-region, small pelagic fish, because of their abundance, partly regulate the trophic dynamics of the upwelling ecosystem in a wasp-waist trophic control^9^. As a result, any change in their abundance might disturb the whole food web, triggering unknown changes in biomass production and species composition, which, in turn, might impact the entire fisheries sector.

## Conclusions

The Cape Blanc, characterised by high productivity and relatively warm waters in a strong upwelling area, emerged as a natural ecological boundary for numerous species. This boundary has witnessed an increasing infiltration of tropical species in the past decades, notably *C. chrysurus, B. auritus,* and *S. guachancho.* The moderate warming observed north of Cape Blanc, particularly over the shelf, bears significant consequences for reshaping the spatial structure of pelagic fish populations, exemplified by the notable changes in *S. aurita* distribution. These changes serve as an early indicator of potential future challenges regarding availability, holding profound implications for the food security of millions of citizens across West Africa. This is especially relevant given the recurrent issues of overfishing capacity and the proliferation of fishmeal/oil factories targeting this species in the region. Our research findings advocate for heightened awareness and urgency for more frequent and regular monitoring and increased research efforts focused on small pelagic in West Africa. Such efforts are essential to understanding the responses of this highly dynamic marine ecosystem, ensuring the sustainability of vital fishery resources, and safeguarding the well-being of coastal communities.

### Supplementary Information

Supplementary Information.

## Data Availability

Part of the data is provided within the manuscript or supplementary information files. Other have to be asked to Abdoulaye Sarre ablaysarrey@yahoo.fr.
