# Supplementary material for: Climate change impacts on small pelagic fish distribution in Northwest Africa: trends, shifts, and risk for food security
Source: Sci Rep. 2024 Jun 3;14:12684. doi: 10.1038/s41598-024-61734-8 (PMC11148102; doi:10.1038/s41598-024-61734-8)
Supplement: Supplementary file 1 — Supplementary Information. [file 41598_2024_61734_MOESM1_ESM.docx]

**Supplementary Sarré *et al*., 2024.**


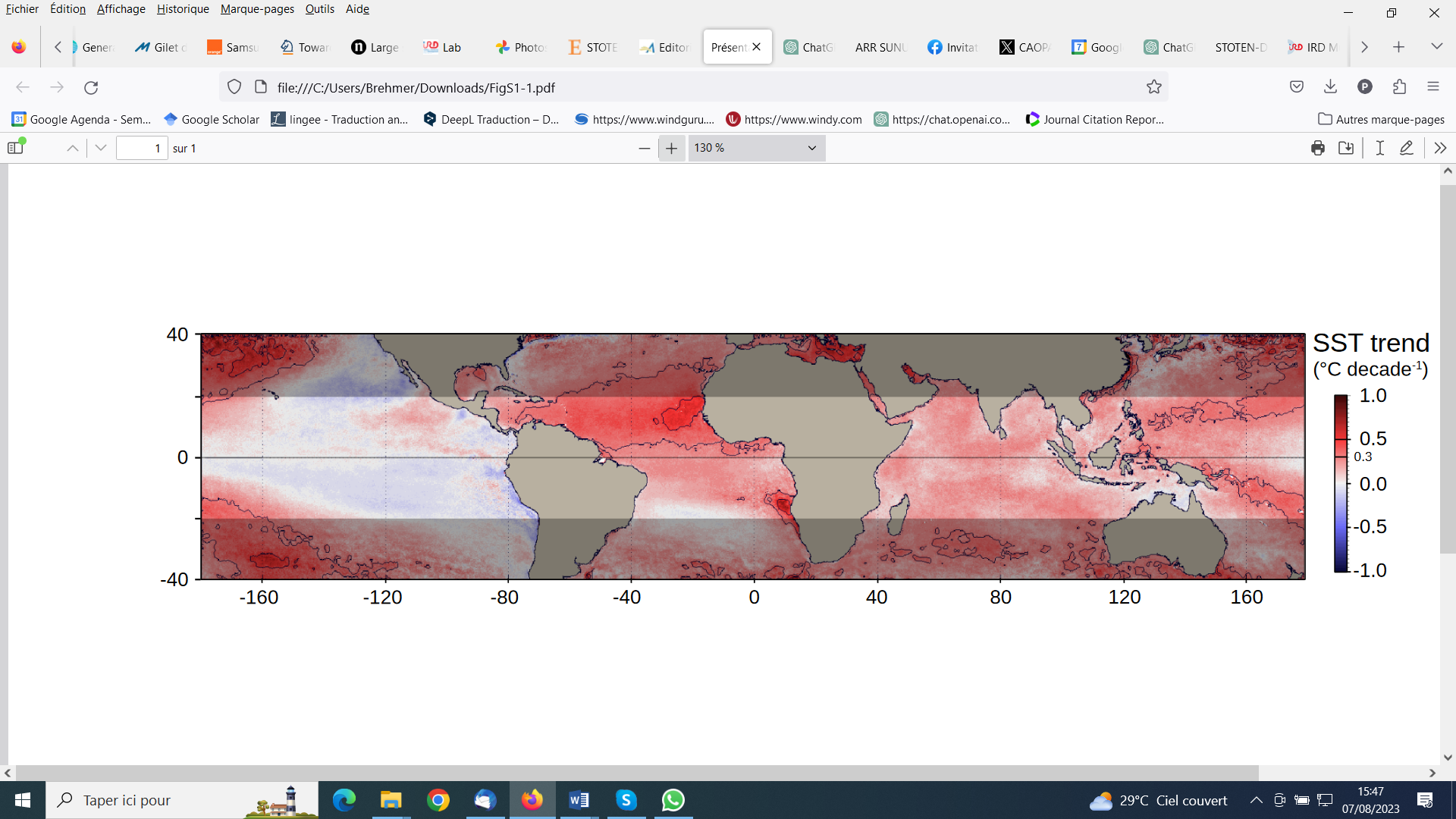


**Fig S1**: Sea Surface Temperature trend in the tropical area showing that West Africa experiences the highest warming in this latitudinal range. The trend is computed for the 36 years 1982-2017 from the Advanced Very-High-Resolution Radiometer (AVHRR) pathfinder v5.3 data set (adapted from Demarcq *et al*. (2018)). Software IDL version 7.1 and Libreoffice 24.2.


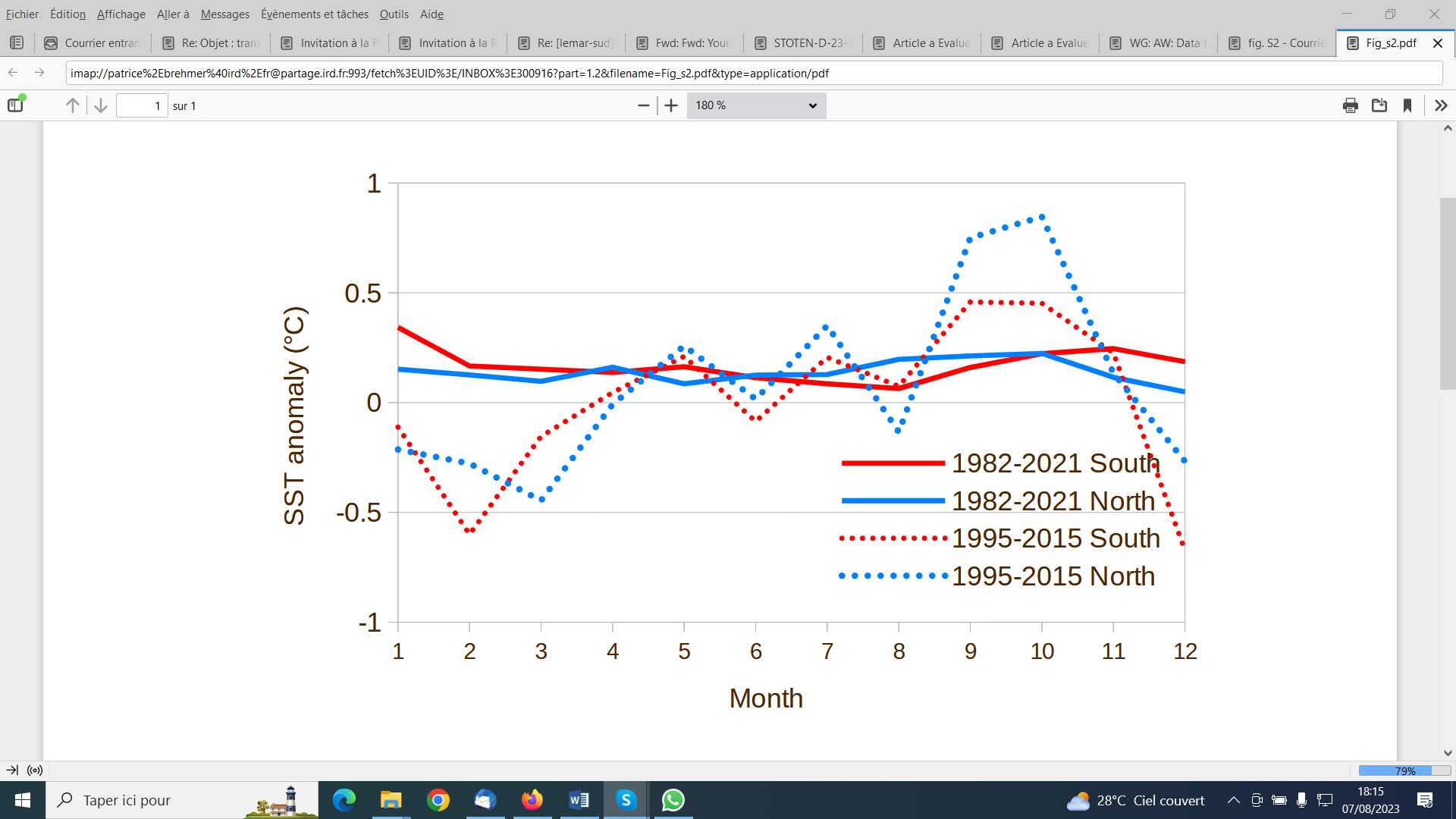


**Fig S2**: Comparative Sea Surface Temperature (SST) monthly anomalies (°C per decade) for the long environmental monitoring (LEM) period (1982-2021, solid lines) and for the biological sampling survey (BSS) period (1995-2015 dotted lines), south and north of Cape Blanc (red and blue lines, respectively), showing that the SST anomaly during the sampling period (centered in November, see Table S1) was similar to the average yearly anomaly, for both periods. Software IDL version 7.1 and Libreoffice 24.2.


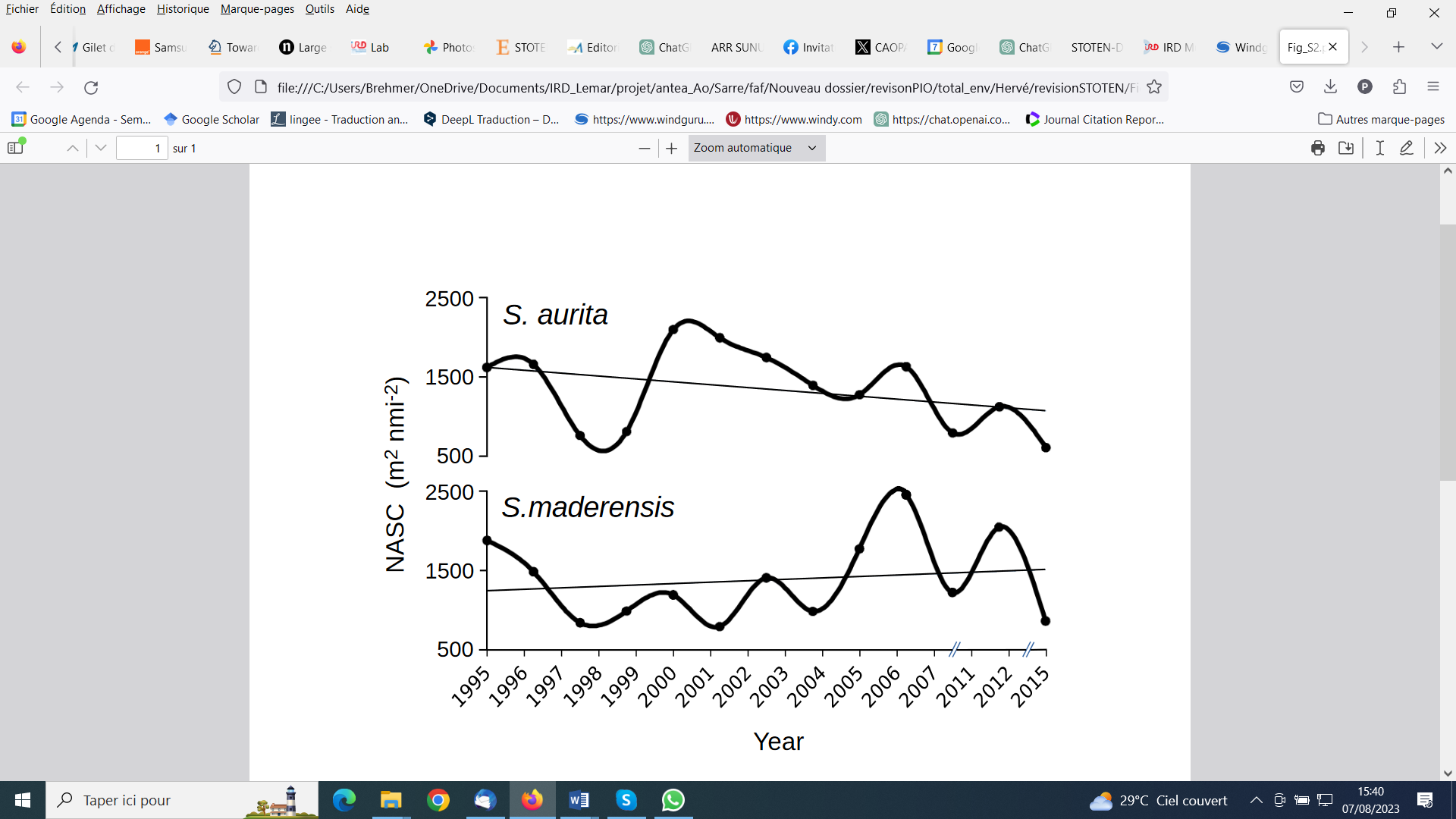


**Fig S3**: Acoustic biomasses of *Sardinella aurita* and *S. maderensis* (expressed in nautical area scattering coefficient ‘NASC’, m^2^ nmi^-2^) obtained from the sea survey annual assessment exercise from 1995-2015 adding 2011 and 2015 carried out by the FRV Dr Fridtjof Nansen in North West African waters (Morocco, Mauritania, Senegal and The Gambia). Software IDL version 7.1 and Libreoffice 24.2.

**Table S1**: List of Nansen annual sea surveys with their starting date in October and end date in December except for 1995 to 1997 (marked by * start day in November). They were all acoustic surveys to assess the small pelagic fish stocks, except in 2011, which was an ecosystemic sea survey characterized by wider spacing between transects (20 nmi).

| Year of sea survey | 1995 | 1996 | 1997 | 1998 | 1999 | 2000 | 2001 |
| --- | --- | --- | --- | --- | --- | --- | --- |
| Start Day (October) | 07* | 01* | 04* | 30 | 30 | 29 | 29 |
| End Day (December) | 14 | 16 | 16 | 15 | 15 | 15 | 14 |
| Year of sea survey | 2002 | 2003 | 2004 | 2005 | 2006 | 2011 | 2015 |
| Start Day (October) | 29 | 25 | 30 | 30 | 31 | 20 | 25 |
| End Day (December) | 15 | 15 | 13 | 1 | 15 | 16 | 14 |

**Table S2**: Northernmost detected latitude (°N) of the main pelagic species sampled during the annual stock assessment surveys, bootstrap of mean comparisons (time series equally divided, n = 10 000) for the period 1995-2015. The significant correlations (*p* < 0.05) are marked in bold.

| Species | Northern limits (°N) | | Northward shift  (km decade^-1^) | *p*-value |
| --- | --- | --- | --- | --- |
|  | 1995-2001 | 2002-2015 |  |  |
| *Sardinella aurita* | 27.2 | 29.1 | **181** | *** |
| *Sardinella maderensis* | 24.0 | 25.2 | 114 | - |
| *Chloroscombrus chrysurus* | 18.5 | 20.4 | **195** | *** |
| *Caranx rhonchus* | 23.8 | 24.5 | **70** | * |
| *Brachydeuterus auritus* | 18.9 | 19.6 | **78** | * |
| *Selene dorsalis* | 18.7 | 19.2 | 43 | - |
| *Trachurus trecae* | 24.4 | 26.3 | **197** | * |
| *Sphyraena guachancho* | 18.2 | 20.1 | **198** | ** |

**Table S3**: Coastal and offshore positions and corresponding shifts (expressed in decimal degree North (°N) or West (°W) according to isotherm main orientations from 1982 to 2021 and their associated spatial shifts (in km per decade), as detailed in Figure 5, providing a visual representation of the transformative trends over the past four decades.

| Sea Surface Isotherm (°C) | Spatial position | | Spatial Shift 1982-2021 (km decade^-1^) | |
| --- | --- | --- | --- | --- |
|  | Coastal | Offshore | Coastal | Offshore |
| 18,5 | 20.0 / 20.4°N | 12.6 / 12.3°W | 40 | 30 |
| 20,0 | 18.5 / 19.7°N | 13.7 / 13.2°W | 125 | 75 |
| 22,0 | 15.5 / 16.5°N | 17.8 / 18.8°N | 125 | 110 |
| 24,0 | 12.7 / 13.1°N | 14.3 / 15.5°N | 45 | 145 |

**Table S4**: Significance of the trends (r Pearson correlation coefficient) of the monthly averages of the environmental parameters (Wind speed, Upwelling index, Chlorophyll-*a* concentration (CHL-*a*), and Sea Surface Temperature (SST)) over the continental shelf displayed in Figure 4 for the full-time series up to 2021 (left) and the 1995-2015 period (right). Significance is summarized as */**/*** for 5, 1, and 0.1% levels, respectively.

|  | Area | r *p*-value ( * / ** / *** ) | | | | | |
| --- | --- | --- | --- | --- | --- | --- | --- |
| Wind speed |  | 1988-2021 | | | 1995-2015 | | |
|  | 1 | 0.679 | 1E-05 | *** | 0.568 | 0.0073 | ** |
|  | 2 | 0.712 | 2E-06 | *** | 0.616 | 0.0030 | ** |
|  | 3 | 0.704 | 3E-06 | *** | 0.744 | 0.0001 | *** |
|  | 4 | 0.889 | 2E-12 | *** | 0.933 | 7E-10 | *** |
|  | 5 | 0.596 | 0.0002 | *** | 0.697 | 0.0004 | *** |
| Upw. Index |  |  |  |  |  |  |  |
|  | 1 | 0.726 | 1E-06 | *** | 0.547 | 0.0103 | ** |
|  | 2 | 0.737 | 7E-07 | *** | 0.647 | 0.0015 | ** |
|  | 3 | 0.724 | 1E-06 | *** | 0.765 | 5E-05 | *** |
|  | 4 | 0.798 | 2E-08 | *** | 0.647 | 0.0015 | ** |
|  | 5 | 0.122 | 0.4925 | - | 0.547 | 0.0103 | ** |
| SST |  | 1982-2021 | | |  | | |
|  | 1 | 0.353 | 0.0257 | * | -0.064 | 0.7827 | - |
|  | 2 | 0.364 | 0.0211 | * | 0.013 | 0.9543 | - |
|  | 3 | 0.525 | 0.0005 | *** | 0.087 | 0.7062 | - |
|  | 4 | 0.514 | 0.0007 | *** | 0.092 | 0.6908 | - |
|  | 5 | 0.475 | 0.0020 | ** | -0.021 | 0.9278 | - |
| CHL-*a* |  | 1998-2021 | | | 1998-2015 | | |
|  | 1 | -0.150 | 0.4828 | - | -0.384 | 0.1161 | - |
|  | 2 | 0.244 | 0.2499 | - | 0.221 | 0.3783 | - |
|  | 3 | -0.290 | 0.1700 | - | -0.242 | 0.3331 | - |
|  | 4 | -0.249 | 0.2414 | - | -0.164 | 0.5144 | - |
|  | 5 | -0.538 | 0.0066 | ** | -0.531 | 0.0235 | * |

**Table S5**: Yearly averages of the monthly data presented in Figure 4: Wind speed (Cross-Calibrated Multi-Platform ‘CCMP’), Ekman Upwelling index, chlorophyll-*a* (Sea-viewing Wide Field of View Sensor ‘SeaWiFS’ and Moderate Resolution Imaging Spectroradiometer ‘MODIS’-corrected) and sea surface temperature (Advanced Very-High-Resolution Radiometer ‘AVHRR’ and MODIS data) from south Morocco (area 1) to Senegal (area 5), respectively, from 1988, 1998 and 1982 up to 2021. Except for the upwelling index, all data are spatially averaged from the coast to 100 km offshore.

| Year/area | Wind speed  (m s^-1^) | | | | | Upwelling index  (m^-3^ s^-1^ m^-1^) | | | | | Chlorophyll-*a*  (mg m^-3^) | | | | | Sea Surface Temperature  (°C) | | | | |
| --- | --- | --- | --- | --- | --- | --- | --- | --- | --- | --- | --- | --- | --- | --- | --- | --- | --- | --- | --- | --- |
|  | 1 | 2 | 3 | 4 | 5 | 1 | 2 | 3 | 4 | 5 | 1 | 2 | 3 | 4 | 5 | 1 | 2 | 3 | 4 | 5 |
| 1982 | - | - | - | - | - | - | - | - | - | - | - | - | - | - | - | 19.07 | 18.61 | 19.34 | 21.45 | 23.74 |
| 1983 | - | - | - | - | - | - | - | - | - | - | - | - | - | - | - | 19.16 | 18.62 | 19.88 | 22.06 | 24.26 |
| 1984 | - | - | - | - | - | - | - | - | - | - | - | - | - | - | - | 19.11 | 18.76 | 19.55 | 21.53 | 23.89 |
| 1985 | - | - | - | - | - | - | - | - | - | - | - | - | - | - | - | 19.30 | 18.82 | 19.74 | 21.56 | 23.68 |
| 1986 | - | - | - | - | - | - | - | - | - | - | - | - | - | - | - | 18.85 | 18.39 | 19.51 | 21.52 | 23.70 |
| 1987 | - | - | - | - | - | - | - | - | - | - | - | - | - | - | - | 19.71 | 18.99 | 20.02 | 22.11 | 24.27 |
| 1988 | 5.96 | 6.64 | 6.57 | 5.29 | 5.07 | 0.58 | 1.15 | 1.09 | 0.70 | 0.87 | - | - | - | - | - | 19.17 | 18.72 | 19.69 | 21.51 | 23.77 |
| 1989 | 5.91 | 6.65 | 6.63 | 5.39 | 5.20 | 0.56 | 1.18 | 1.17 | 0.79 | 0.97 | - | - | - | - | - | 19.31 | 18.72 | 19.74 | 21.72 | 23.94 |
| 1990 | 5.64 | 6.53 | 6.66 | 5.50 | 4.90 | 0.54 | 1.12 | 1.11 | 0.76 | 0.84 | - | - | - | - | - | 19.49 | 18.84 | 19.93 | 21.92 | 24.19 |
| 1991 | 6.03 | 6.82 | 6.77 | 5.33 | 5.01 | 0.57 | 1.19 | 1.13 | 0.73 | 0.90 | - | - | - | - | - | 19.03 | 18.49 | 19.47 | 21.43 | 23.58 |
| 1992 | 5.98 | 6.89 | 6.76 | 5.38 | 4.91 | 0.58 | 1.22 | 1.12 | 0.73 | 0.82 | - | - | - | - | - | 19.03 | 18.44 | 19.57 | 21.53 | 23.76 |
| 1993 | 6.09 | 7.07 | 6.92 | 5.54 | 5.07 | 0.59 | 1.26 | 1.16 | 0.79 | 0.91 | - | - | - | - | - | 19.07 | 18.51 | 19.35 | 21.51 | 24.07 |
| 1994 | 6.14 | 7.13 | 6.75 | 5.33 | 4.94 | 0.64 | 1.28 | 1.09 | 0.68 | 0.79 | - | - | - | - | - | 19.12 | 18.59 | 19.86 | 21.82 | 23.87 |
| 1995 | 6.01 | 6.74 | 6.54 | 5.26 | 4.94 | 0.61 | 1.15 | 1.01 | 0.66 | 0.82 | - | - | - | - | - | 19.68 | 19.26 | 20.47 | 22.26 | 24.35 |
| 1996 | 6.13 | 6.81 | 6.71 | 5.40 | 4.85 | 0.63 | 1.20 | 1.14 | 0.76 | 0.82 | - | - | - | - | - | 19.63 | 19.32 | 20.35 | 22.16 | 24.35 |
| 1997 | 5.66 | 6.40 | 6.28 | 4.99 | 4.40 | 0.51 | 1.02 | 0.95 | 0.62 | 0.63 | - | - | - | - | - | 20.16 | 19.38 | 20.20 | 22.27 | 24.56 |
| 1998 | 6.37 | 6.79 | 6.39 | 5.19 | 4.78 | 0.67 | 1.18 | 1.02 | 0.67 | 0.75 | 1.80 | 3.75 | 6.21 | 7.81 | 7.14 | 19.56 | 19.15 | 20.35 | 22.32 | 24.70 |
| 1999 | 6.72 | 7.51 | 7.19 | 5.46 | 5.02 | 0.75 | 1.42 | 1.21 | 0.70 | 0.79 | 2.41 | 3.40 | 5.14 | 3.88 | 4.35 | 19.14 | 18.52 | 19.91 | 21.92 | 23.96 |
| 2000 | 6.46 | 7.35 | 7.16 | 5.59 | 4.96 | 0.72 | 1.39 | 1.28 | 0.74 | 0.77 | 2.11 | 3.13 | 5.47 | 4.04 | 6.18 | 19.19 | 18.68 | 19.98 | 22.00 | 24.36 |
| 2001 | 6.52 | 7.24 | 7.14 | 5.55 | 4.94 | 0.72 | 1.38 | 1.31 | 0.74 | 0.75 | 2.63 | 3.59 | 4.63 | 4.07 | 5.28 | 19.34 | 18.94 | 20.60 | 22.82 | 24.74 |
| 2002 | 6.66 | 7.22 | 7.07 | 5.68 | 5.18 | 0.71 | 1.33 | 1.25 | 0.79 | 0.85 | 2.27 | 4.40 | 7.68 | 4.38 | 5.25 | 19.53 | 19.11 | 20.27 | 22.23 | 24.21 |
| 2003 | 6.45 | 7.15 | 7.09 | 5.63 | 5.13 | 0.71 | 1.34 | 1.29 | 0.78 | 0.86 | 2.48 | 4.85 | 6.81 | 5.65 | 5.38 | 19.52 | 18.82 | 19.81 | 21.83 | 24.00 |
| 2004 | 6.60 | 7.06 | 6.92 | 5.67 | 5.18 | 0.71 | 1.27 | 1.20 | 0.80 | 0.87 | 1.90 | 4.37 | 7.54 | 4.57 | 4.18 | 19.59 | 19.18 | 20.22 | 22.17 | 24.20 |
| 2005 | 6.50 | 7.22 | 7.13 | 5.80 | 5.13 | 0.72 | 1.34 | 1.29 | 0.85 | 0.82 | 1.76 | 3.45 | 6.27 | 4.48 | 3.24 | 19.50 | 19.06 | 20.48 | 22.69 | 24.86 |
| 2006 | 6.57 | 7.34 | 7.19 | 5.86 | 5.02 | 0.70 | 1.38 | 1.26 | 0.80 | 0.77 | 1.78 | 3.31 | 5.90 | 5.76 | 4.38 | 19.73 | 19.41 | 20.66 | 22.27 | 24.37 |
| 2007 | 6.49 | 7.25 | 7.27 | 6.11 | 5.22 | 0.69 | 1.36 | 1.30 | 0.90 | 0.84 | 1.48 | 3.27 | 5.63 | 6.12 | 4.98 | 19.42 | 18.92 | 19.93 | 21.98 | 24.26 |
| 2008 | 6.84 | 7.33 | 7.25 | 6.03 | 5.17 | 0.76 | 1.39 | 1.30 | 0.86 | 0.84 | 1.71 | 3.46 | 4.70 | 4.74 | 5.48 | 19.33 | 19.03 | 20.19 | 22.34 | 24.65 |
| 2009 | 6.39 | 7.13 | 7.34 | 6.16 | 5.28 | 0.68 | 1.35 | 1.37 | 0.97 | 0.95 | 2.35 | 3.86 | 5.16 | 4.28 | 4.76 | 19.58 | 19.25 | 20.47 | 22.16 | 24.16 |
| 2010 | 6.31 | 6.84 | 6.76 | 5.86 | 5.06 | 0.66 | 1.24 | 1.26 | 0.90 | 0.84 | 1.37 | 3.19 | 5.03 | 4.37 | 2.91 | 20.25 | 20.18 | 21.23 | 23.01 | 24.95 |
| 2011 | 6.46 | 7.44 | 7.46 | 6.34 | 5.29 | 0.72 | 1.40 | 1.39 | 0.96 | 0.93 | 1.72 | 3.95 | 5.82 | 4.60 | 4.34 | 19.65 | 19.25 | 20.40 | 22.30 | 24.37 |
| 2012 | 6.64 | 7.35 | 7.22 | 6.33 | 5.32 | 0.76 | 1.38 | 1.23 | 0.88 | 0.87 | 1.80 | 4.42 | 6.04 | 4.99 | 4.68 | 19.39 | 18.96 | 20.15 | 22.02 | 24.14 |
| 2013 | 6.56 | 7.36 | 7.38 | 6.15 | 5.14 | 0.71 | 1.40 | 1.35 | 0.90 | 0.79 | 1.68 | 3.82 | 6.26 | 5.15 | 4.49 | 19.56 | 19.16 | 20.39 | 22.46 | 24.63 |
| 2014 | 6.72 | 7.54 | 7.58 | 6.35 | 5.25 | 0.72 | 1.45 | 1.43 | 0.96 | 0.85 | 1.95 | 3.94 | 4.95 | 4.06 | 3.84 | 19.36 | 18.73 | 19.97 | 22.02 | 24.13 |
| 2015 | 6.68 | 7.43 | 7.34 | 6.16 | 5.09 | 0.74 | 1.41 | 1.31 | 0.86 | 0.76 | 2.25 | 4.51 | 5.29 | 4.94 | 4.55 | 19.44 | 18.82 | 20.24 | 22.24 | 24.30 |
| 2016 | 6.63 | 7.20 | 7.05 | 6.20 | 5.14 | 0.71 | 1.33 | 1.24 | 0.88 | 0.82 | 2.27 | 4.00 | 5.60 | 4.45 | 4.24 | 19.58 | 19.10 | 20.38 | 22.34 | 24.50 |
| 2017 | 6.42 | 7.01 | 7.04 | 6.21 | 5.07 | 0.66 | 1.23 | 1.20 | 0.86 | 0.79 | 1.85 | 4.39 | 6.05 | 5.13 | 4.08 | 19.72 | 19.37 | 20.45 | 22.49 | 24.54 |
| 2018 | 6.83 | 7.56 | 7.53 | 6.36 | 5.32 | 0.76 | 1.48 | 1.44 | 0.96 | 0.95 | 1.73 | 3.26 | 4.82 | 4.71 | 3.75 | 19.19 | 18.64 | 19.64 | 21.51 | 23.69 |
| 2019 | 6.51 | 7.44 | 7.26 | 6.15 | 5.33 | 0.72 | 1.44 | 1.35 | 0.89 | 0.90 | 2.12 | 3.88 | 5.75 | 4.66 | 5.43 | 19.18 | 18.76 | 20.05 | 21.91 | 24.10 |
| 2020 | 6.27 | 7.28 | 6.96 | 6.08 | 5.34 | 0.70 | 1.40 | 1.22 | 0.87 | 0.87 | 2.29 | 3.54 | 4.83 | 3.95 | 3.48 | 19.62 | 19.03 | 20.64 | 22.67 | 24.77 |
| 2021 | 6.38 | 7.51 | 7.26 | 6.19 | 5.23 | 0.72 | 1.50 | 1.40 | 0.95 | 0.89 | 2.03 | 4.84 | 5.79 | 4.34 | 3.66 | 19.23 | 18.69 | 19.76 | 21.90 | 24.26 |

**Reference Supplementary material**

Demarcq Hervé, Sarré Abdoulaye, Kouassi Aka Marcel, Uanivi Uatjavi, Jeyid Mohamed Ahmed, El Ayoubi S., Bamy Idriss Lamine, Brehmer Patrice. (2018). Spatial Environmental trends in the three Atlantic African Large Marine Ecosystems in a context of global warming. 17 – 20 April, PREFACE international Conference on Ocean, Climate and Ecosystems, Arrecife, Lanzarote Canary Islands, Spain <https://hal.archives-ouvertes.fr/hal-02779826v1>
